# Imaging features of periostitis as a manifestation of IgA vasculitis: A case report

**DOI:** 10.1097/MD.0000000000022480

**Published:** 2020-09-25

**Authors:** Ji Hoon Noh, Bo Mi Chung, Wan Tae Kim

**Affiliations:** a Department of Radiology, Veterans Health Service Medical Center, 53, Jinhwangdo-ro 61-gil, Gangdong-gu; b Departement of Radiology, Graduate School of Chung-Ang University, Seoul, Republic of Korea.

**Keywords:** IgA vasculitis, periosteal reaction, periostitis, systemic vasculitis

## Abstract

**Introduction:**

Periostitis in systemic vasculitis is very rare with only a few previously reported cases. The reported cases were seen in polyarteritis nodosa or Takayasu arteritis. We report the first case of periostitis associated with IgA vasculitis with demonstration of computed tomography (CT), magnetic resonance imaging (MRI) features, and serial changes of radiographs.

**Patient concerns:**

A 74-year-old man visited an orthopedic outpatient clinic for pain in both lower legs and left ankle pain. He underwent a total ankle arthroplasty of the left ankle 3 years ago. His medical history disclosed IgA vasculitis/nephropathy caused by cephalosporin antibiotic class 5 months earlier. Plain radiography, MRI of the right lower leg, CT scan of the left ankle showed single lamellar to spiculated periosteal reactions at both tibia, fibula and left calcaneus. There was no evidence of bone or soft tissue mass lesions or cortical destruction.

**Diagnosis:**

We concluded that this was a case of periosteal reactions associated with IgA vasculitis for the following reasons: (1) periosteal biopsy was negative for tumor. (2) there was no pulmonary lesion on chest radiography and CT, no history of trauma, inflammatory arthritis, metabolic disease, or genetic disease that could cause periostitis at multiple bones, (3) the initial MRI showed predominant signal changes around the tibial and fibular shafts, possibly explaining subsequent periosteal reactions, and (4) symptoms subsided with conservative treatment and follow-up radiographs showed remodeling of periosteal reactions.

**Interventions:**

The patient took conservative management.

**Outcomes:**

His calf pain improved, and a radiograph 7 months later showed remodeling to the solid or single lamellar periosteal reaction along the both tibia and left fibula.

**Conclusion:**

Awareness of this uncommon manifestation would help differential diagnosis of periostitis and could help decrease the patient's anxiety. It should also be noted that periosteal reactions by benign entities could cause aggressive-looking periosteal reactions in post-operative regions.

## 1 Introduction

Periosteal reactions at multiple bones are nonspecific pathologies with many causes, including tumors, infections, trauma, metabolic diseases, inflammatory diseases, and rarely, vascular causes.^[1]^ Periosteal reactions as manifestations of systemic vasculitis are uncommon, with only a few case reports in the English literature.^[2–9]^ Polyarteritis nodosa (PAN) was the most common type of vasculitis presenting periostitis, followed by Takayasu arteritis (TA).^[2–9]^ Here, we report the first case of periostitis associated with IgA vasculitis with demonstration of computed tomography (CT), magnetic resonance imaging (MRI) features, and serial changes of radiographs in a 74-year-old man who presented with pain in both lower legs. When multifocal periosteal reactions are encountered, considering systemic vasculitis as a potential cause could help with differential diagnosis.

## 2 Case report

A 74-year-old man visited an orthopedic outpatient clinic for pain in both lower legs and left ankle pain. Physical examination at this presentation revealed mild swelling around the left ankle. No heating sensation, redness, or tenderness was noted in either lower leg or ankle. He was afebrile and his vitals were a pulse rate of 85 beats per minute and blood pressure of 71/119 mm Hg. Laboratory examinations were only notable for erythrocyte sedimentation rate of 36 mm/h, creatinine of 1.7 mg/dL, and blood urea nitrogen of 23 mg/dL. He had a history of a total ankle arthroplasty of the left ankle 3 years ago.

His medical history disclosed IgA vasculitis. He had been admitted to our institution 5 months previously, presenting with pain, swelling, and petechia in both legs. He took cephalosporin antibiotic class for the impression of cellulitis 3 days before. Laboratory examinations revealed an elevated white blood cell count of 13390/mm^3^, C-reactive protein of 173.3 mg/L, and erythrocyte sedimentation rate of 120 mm/h. Serological studies were positive for IgA (516 mg/dL). Biopsy from the skin lesions showed features of vasculitis with predominant IgA deposits at the small vessel walls. Urinalysis revealed hematuria and proteinuria. Renal biopsy showed severe interstitial fibrosis and global sclerosis. The diagnosis of IgA vasculitis/nephropathy caused by cephalosporin antibiotic class was made based on clinical features and biopsy findings. He was given an oral corticosteroid with follow-up visits in the outpatient nephrology clinic after discharge at the time of presentation.

**Figure 1:**
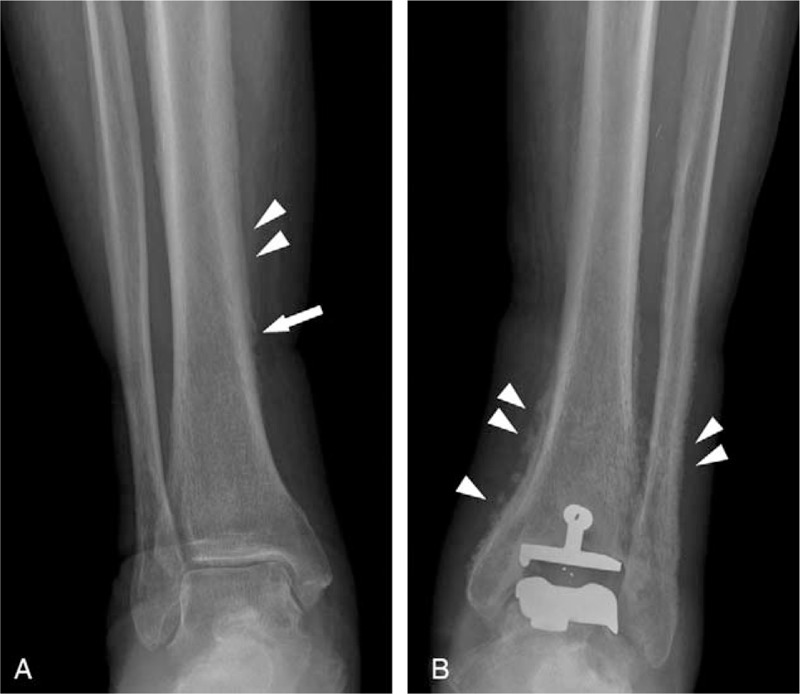
(A) Frontal radiograph of the right lower leg demonstrates a single lamellar periosteal reaction at the tibial shaft (arrowheads). A focal irregular periosteal reaction is also noted (arrow). (B) Frontal radiograph of the left lower leg shows spiculated and thick irregular periosteal reactions at the distal tibia and fibula. Note total ankle arthroplasty state at the left ankle (arrowheads).

**Figure 2:**
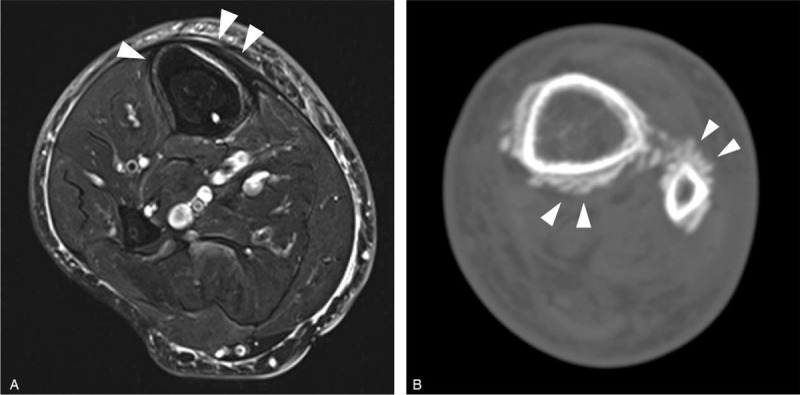
(A) Axial fat-suppressed T2-weighted image of the right lower leg demonstrates a periosteal thickening with mineralization at the anterior aspect of the tibia, suggesting a single lamellar periosteal reaction (arrowheads). (B) CT of the left ankle demonstrates spiculated periosteal reactions at the distal tibia and fibula (arrowheads). No bone or soft tissue mass lesions or cortical destruction were noted.

Plain radiography showed a single lamellar periosteal reaction in the right tibial shaft (Fig. 1A). There were spiculated or thick irregular periosteal reactions at the left distal tibia and fibula (Fig. 1B). An MRI of the right lower leg revealed a single lamellar periosteal reaction in the tibial shaft (Fig. 2A). A CT scan of the left ankle showed spiculated periosteal reactions at the distal tibia, fibula, and calcaneus (Fig. 2B). There was no evidence of bone or soft tissue mass lesions or cortical destruction.

**Figure 3:**
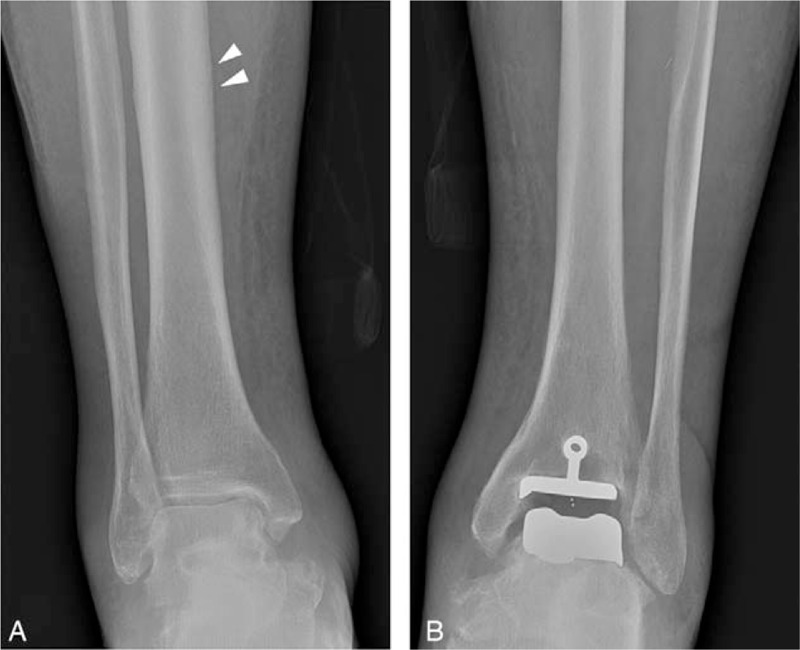
Frontal radiographs of the right (A) and left (B) lower leg 5 months earlier. There is a single lamellar periosteal reaction at the medial border of the right tibial shaft (arrowheads), which is thinner than the plain radiography in Figure 1. No bony abnormalities, except post-operative changes of the left ankle, were noted in the left lower leg. Note soft tissue swelling in both lower legs.

**Figure 4:**
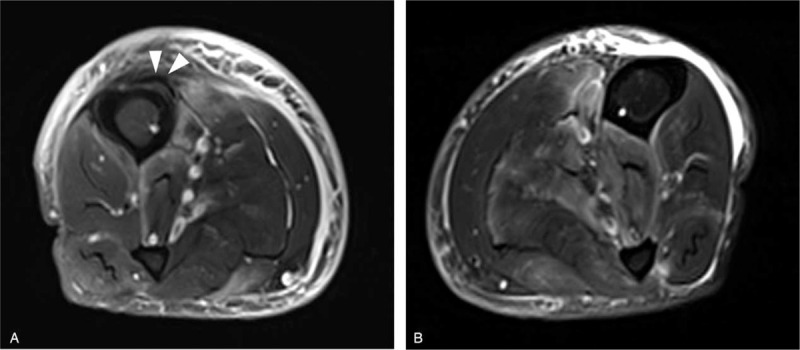
Axial fat-suppressed T2-weighted images of the right (A) and left (B) lower leg 5 months earlier. The right lower leg MRI shows periosteal elevation with mineralization at the tibial shaft (arrowheads). No periosteal reactions were noted in the left tibia or fibula. There are multifocal patchy edema in both lower leg muscles, especially prominent around both tibia, left fibula, and both intermuscular septa. Note diffuse swelling of the subcutaneous layers in both legs.

We reviewed previous imaging studies performed 5 months ago, when the patient presented with pain, swelling, and petechia in both legs. Radiography of both lower legs revealed soft tissue swelling in both of the lower legs, a single lamellar periosteal reaction in the right tibial shaft, and normal left tibial and fibular shafts (Fig. 3). MRI of the right lower leg revealed a single lamellar periosteal reaction at the anterior aspect of the tibial shaft (Fig. 4A). MRI of both lower legs showed multifocal patchy signal changes in the muscles of both lower legs, especially prominent around the tibia and fibula, and intermuscular septa, findings which were initially interpreted as muscle involvement of systemic vasculitis (Fig. 4A and B).

**Figure 5:**
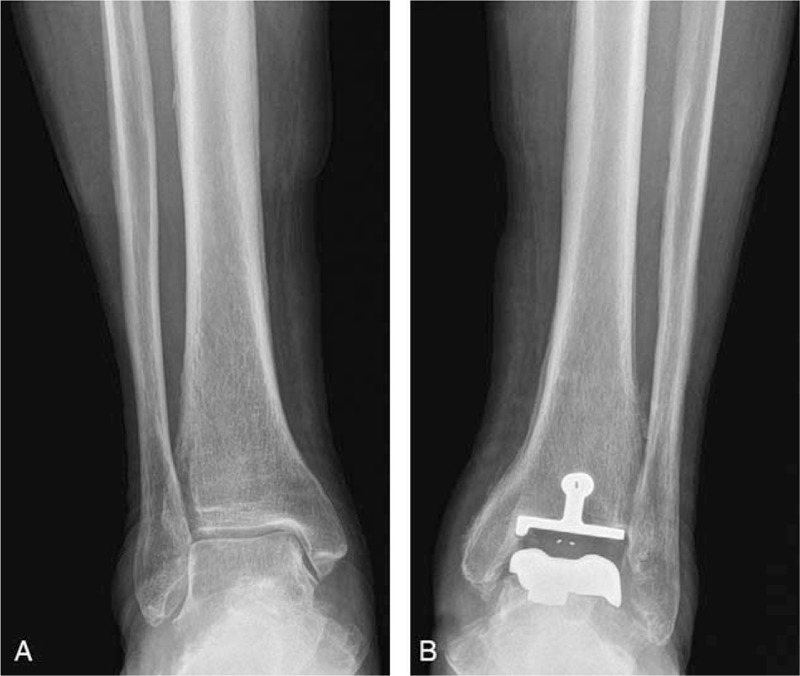
Follow-up frontal radiographs of the right (A) and left (B) lower legs taken 7 months later show thick single lamellar or solid periosteal reactions in both tibias and in the left fibula.

A periosteal biopsy was performed to exclude unusual tumor of periosteal origin because periosteal reaction at left distal tibia seemed aggressive. The histopathological examination revealed bone fragments and some inflammatory cells and was negative for tumor. There was no pulmonary lesion on chest radiography and CT. He had no history of trauma, inflammatory arthritis, metabolic disease, or genetic disease that could cause periostitis at multiple bones. We concluded these lesions as periostitis associated with IgA vasculitis. His calf pain improved after conservative management and a radiograph 7 months later showed remodeling to the solid or single lamellar periosteal reaction along the both tibia and left fibula (Fig. 5).

This study received institutional review board approval and written informed consent was obtained from the patient for publication of this article.

## 3 Discussion

IgA vasculitis, formerly known as Henoch-Schonlein purpura, is an immune complex-mediated, small vessel vasculitis with predominant IgA deposits in the vessel wall.^[10]^ IgA vasculitis is the most common vasculitis in childhood. It is less common in adults, with an annual incidence of 0.1 to 1.8 per 1,00,000 individuals.^[11]^ IgA vasculitis is characterized clinically by palpable purpura, gastrointestinal, renal, or joint involvement.^[10]^ Although arthritis or arthralgia is known as a musculoskeletal manifestation, there is no case of periostitis associated with IgA vasculitis. We concluded that this was a case of periosteal reactions associated with IgA vasculitis for the following reasons: (1) periosteal biopsy was negative for tumor. (2) there was no pulmonary lesion on chest radiography and CT, no history of trauma, inflammatory arthritis, metabolic disease, or genetic disease that could cause periostitis at multiple bones, (3) the initial MRI showed predominant signal changes around the tibial and fibular shafts, possibly explaining subsequent periosteal reactions, and (4) symptoms subsided with conservative treatment and follow-up radiographs showed remodeling of periosteal reactions.

Periosteal reactions as manifestations of systemic vasculitis are uncommon, with a small number of case reports in the English literature.^[2–9]^ PAN was the most common type of vasculitis presenting periostitis, followed by TA.^[2–9]^ Although the mechanism of periosteal reactions in systemic vasculitis is uncertain, researchers have suggested several hypotheses. McConachie et al^[9]^ stated that arterial insufficiency appeared to be the main mechanism, a theory questioned by Kim et al^[8]^ proposed that periosteal reactions may occur as an inflammatory process independent of direct vessel involvement or hypoxia, because periosteal reactions occurred in normoxic regions in their patient. Bogaert et al^[4]^ suggested a common inflammatory process of both vessels and periosteal bone as a mechanism of periosteal reactions based on their MRI findings that revealed edema at intermuscular regions and near intermuscular vessels. In a case report of PAN presenting as periostitis, the MRI showed high SI and enhancement around the periosteum of tibia and intermuscular septum.^[6]^ Similar to previous cases,^[4,6]^ the initial MRI in our case showed predominant signal changes around the periosteum and intermuscular septum, which were thought to reflect inflammation of the periosteum and soft tissues around the small vessels that led to periosteal reactions. Small vessel vasculitis at and around the periosteum would have resulted in vascular damage and caused tissue hypoxia and local production of bone growth factors.

Periosteal reactions by benign causes generally show solid or single lamellar patterns.^[1]^ Spiculated patterns are usually associated with the worst prognosis, as they occur in aggressive and fast-growing diseases.^[1]^ Our case showed spiculated periosteal reactions at the left lower leg, a finding which led to a periosteal biopsy. We speculated that the previous total ankle arthroplasty would have altered structures and vascularity of the periosteum around the ankle, causing more active reactions to inflammation or hypoxia of the periosteum. Similarly, in a report by Cheon et al, a 10-year-old boy with TA presented periosteal reactions with thick, fluffy margins, because the periosteum is more active in children.^[2]^

In conclusion, this is the first case report of periostitis associated with IgA vasculitis in an adult. Awareness of this uncommon manifestation would help differential diagnosis of periostitis and could help decrease the patient's anxiety. It should also be noted that periosteal reactions by benign entities could cause aggressive-looking periosteal reactions in post-operative regions.

## Author contributions

**Data collection:** Ji Hoon Noh.

**Case analysis:** Bo Mi Chung.

**Writing_original draft:** Ji Hoon Noh, Bo Mi Chung.

**Writing_review, editing:** Bo Mi Chung.

**Supervision:** Wan Tae Kim.
